# 2326: Successful hand function recovery after stroke

**DOI:** 10.1017/cts.2017.222

**Published:** 2018-05-10

**Authors:** Shashwati Geed, Peter S. Lum, Michelle L. Harris-Love, Jessica Barth, Peter E. Turkeltaub, Alexander W. Dromerick

**Affiliations:** Georgetown - Howard Universities, Washington, DC, USA

## Abstract

OBJECTIVES/SPECIFIC AIMS: Upper-extremity (UE) impairment affects 88% of stroke survivors due to dysfunctional shoulder-hand coordination. Patients may be able to grasp with the arm at rest, but unable to grasp in a functional context (eg, from a high shelf) because shoulder use elicits involuntary hand muscle activity. Further, much rehabilitation research is directed at unsuccessful stroke recovery (patients with persistent UE impairment) but very little towards patients who show successful clinical recovery (such as those with mild UE impairment) even though these patients have attained the desired rehabilitation outcome. We examined the neurophysiological trajectory of successful compared to unsuccessful post-stroke recovery in the context of functional UE movements to clearly identify what factors are necessary for successful recovery of functional UE movements after stroke. METHODS/STUDY POPULATION: We studied 3 populations: (1) mildly-impaired patients, early (at <17 d, 30 d, 90 d, and 180 d) after stroke as a model of successful post-stroke recovery, (2) moderately-impaired, chronic patients (>6-months post stroke) with persistent hand function impairment, as a model of incomplete post-stroke recovery (unsuccessful recovery), and (3) Healthy age-range matched controls. We used transcranial magnetic stimulation (TMS) in all 3 groups at the given time points to measure corticomotor excitability (motor evoked potentials, recruitment curve), corticomotor inhibition (short-interval intracortical inhibition, long-interval intracortical inhibition), and intracortical facilitation of hand muscles with the shoulder positioned in different degrees of flexion or abduction (these shoulder positions are known to elicit involuntary, undesired hand muscle activation, which leads to UE dysfunction and impairment in individuals with stroke). RESULTS/ANTICIPATED RESULTS: Data collection are in process and will be presented. Preliminary data from controls shows that corticomotor excitability of selected hand muscles is affected by changes in shoulder position. Preliminary findings in controls are consistent with clinical findings in stroke that certain shoulder positions elicit involuntary and undesired hand muscle activation, leading to UE dysfunction and disability. Findings from the stroke groups will be presented. DISCUSSION/SIGNIFICANCE OF IMPACT: We hypothesize that this centrally-facilitated coupling between shoulder and hand muscles is disrupted after stroke, which may play a central role in the inability of patients to perform functional UE movements. By comparing the TMS metrics in mildly-impaired Versus moderately-impaired chronic patients, we will be able to identify the longitudinal change in neurophysiology underlying shoulder-hand coordination that is associated with successful or unsuccessful clinical recovery of UE function after stroke. Thus, these findings will help us distinguish between the neurophysiology underlying successful from unsuccessful UE recovery leading to more mechanism-based interventions for UE dysfunction post stroke in the future.

